# Supplementary material for: Transfection types, methods and strategies: a technical review
Source: PeerJ. 2021 Apr 21;9:e11165. doi: 10.7717/peerj.11165 (PMC8067914; doi:10.7717/peerj.11165)
Supplement: Supplemental Information 1 — Cell lines details: AGS: Human gastric adenocarcinoma cell line; A549: Human adenocarcinomic alveolar basal epithelial cell; A673: Human ewing sarcoma cell line; A-10 SMCs: Rats smooth muscle cells; bMDM: Bovine monocyte-derived macrophages; C2C12: Mouse myoblasts; C3H10T1/2: Mouse stem cells; HAECs: Human aorta endothelial cells; HASMCs: Human aorta smooth muscle cells; hBM-MSC: Human bone marrow mesenchymal stem cells; HCT116: Human colorectal carcinoma cell line; HEK: Primary human epidermal keratinocytes; HEK293: Human embryonic kidney cells 293; HeLa: Human cervical cancer cell; Hep G2: Human hepatocellular carcinoma cells; hiPSC-CMS: human induced pluripotent stem cells derived cardiomyocytes; HL-60: Human leukemia cell line; hPDLSCs: Human periodontal-ligament stem cells; hTERT MSCs: Human telomerase reverse transcriptase mesenchymal stem cells; Huh-7: Human liver cell line; HUVECs: Human umbilical cord vein cells; JU77: Human primary myoblast and human lung mesothelioma cells; MCF-7: Oestrogen responsive breast cancer cell line; MC3T3-E1: Mouse preosteoblasts; MDA-MB-231: Triple negative breast cancer cell line; MESCs: Sickle cell disease transgenic mice embryonic stem cell; Neuro2a: mice neuroblastoma cells; Panc 04.03: Human pancreas epithelial cancer cell line; PECs: Primary endothelial cells; PTE and THE: Primary pig and human tracheal epithelial cells; PT-30: Human epithelial precancer cells ; P16: Primary embryonic pig fibroblasts; REF: Embryonic rabbit ear fibroblasts; SH-SY5Y: Human neuroblastoma cells; SKOV3: Human ovarian cancer cell line; SMA: Spinal muscular atrophy fibroblast cells; UMR: Rat osteosarcoma cell line; U87MG: human glioblastoma cells ; Z3: Embryonic zebrafish cell line; 4T1: Mouse mammary carcinoma; 16HBE14o- and CFBE41o-: Immortalized human bronchial epithelial cells. Abbreviations for transfection related chemicals/methods: AT: Attractene; DAC: 3β-[N-(N,N’-dimethylaminoethane)-carbamoyl]; DC: 3β-[(N,N’-dimethylaminoethane)-carbamo [file peerj-09-11165-s001.docx]

**Supplementary Table 1: Comparison of the transfection efficiencies of commercially available transfection reagents and transfection conditions reported in different studies (n=20)**

| **Type of nuclei acid of interest** | **Type of cell lines used** | **Brief description of the transfection protocols** | **Type of the plasmid / oligonucleotides used** | **Comparison of the transfection efficiencies between different reagents / methods used** | **Other additional information (s)** | **References** |
| --- | --- | --- | --- | --- | --- | --- |
| DNA | PECs isolated from HUVECs | - 1.2 to 3 x 10^5^ cells / well (6-wells plate). - All cells were below passage number 6. - 0.2-2.5 µg DNA and 6-15 µL of different transfection reagents were used. - Transfection reaction mixture was incubated for 3 to 5 hours. | DNA plasmids carrying green fluorescent protein (eGFP) | EFT > FG6 > DTP  FG6 was the least toxic reagent, evidenced by the presence of least number of detached cells. | - Effect of temperature on the transfection efficiency:   37^0^C > 22^0^C > 6^0^C (p<0.0001)   - Fluorescence microscopy was used to assess transfection efficiency. | (Young *et al.* 2002) |
| DNA | HASMCs, HAECs and A-10 SMCs | - In 6-wells plate, 7 x 10^4^ cells / well were seeded. - 0.5 to 2 µg DNA was mixed with different volume of transfection reagents at different ratio and incubated for 3 to 5 hours. - Transfection efficiency was measured at 48-hours post-transfection. | pAH7-EGFP plasmid | For **HAEC**,  SFT > DAC-30 > EFT = LP+ve = LF = DC-30 > FG6  For **HASMC**,  FG6 > SFT > DAC-30 > LP+ve > LF > DC-30 > EFT  For **A-10 SMC**,  FG6 > DAC-30 > SFT > DC-30 > LF > EFT > LP+ve | - Flow cytometry assessed transfection efficiency. - In A-10 SMC, HASMC and HAFC, SFT produced highest cytotoxicity effect, followed by DAC-30 and LP+ve. - DAC-30 is the best transfection reagent with acceptable toxicity. | (Kiefer *et al.* 2004) |
| DNA | Primary human myoblasts cells from skeletal biopsies | - 2.1 x 10^5^ cells per 3.5cm plate. - For FG6, the reagent (µL) used ranged from 2 to 6 whereas the DNA (µg) used ranged from 0.5 to 4. For EFT, the reagent (µL): DNA (µg) ratio ranged from 10:1 to 50:1. - For EG500, the quantity of the reagent used ranged from 3 to 12.5eq. Transfection efficiency was measured at 24-hours post-transfection. | DNA plasmid (pEGFP-C3/SMN) carrying *survival motor neuron (SMN)* gene | FG6 > EFT > *EG500  *EG500 is a PEI.  In overall, FG6 is the best transfection reagent. | - Transfection efficiency monitoring using fluorescence-activated cell sorting (FACS). - Cell cytotoxicity comparison:   EG500 > FG6 > EFT   - Transfection efficiency is lower in primary cell lines (<20%). | (Arnold *et al.* 2006) |
| siRNA | 11 cell types, which included HeLa, HEK293, SKOV3, A549, HCT116, Huh7, A673, XMD5, UMR, MDA-MB-231 and Panc 4.03 | - 25nM of the siRNA in 4 µL of the Opti-MEM reagent. - Cells number used ranged from 750 to 5000 cells in each well of a 384-wells plate. - The transfection reagents used ranged from 0.03 to 0.28 µL / well. - SiRNA-lipid complexes were incubated for 0.5 hours before added to the cells. | siRNA | For **SKOV3**,  DFT2 > DFT4 > OF > HPT  The transfection efficiencies in other cell lines were not further shown/displayed.  OF was the safest reagent but did not produce best transfection efficiency.  DFT4 was the reagent that produced best transfection efficiencies but associated with high toxicities in most cell lines. | - GFP and luciferase-based reporting system to analyse transfection efficiency. - DFT2 was concluded as the best among all tested reagents. | (Borawski *et al.* 2007) |
| DNA | HUVECs | - 1 – 2 x 10^5^ HUVEC cells / well were seeded in 6 wells plate. - Different ratio of the transfection reagent (µL) to DNA (µg) were prepared for each transfection reagent type. - Transfection efficiency was assessed at 24- and 48-hours. | - Plasmids containing enhanced green fluorescent protein (pEFGP-N1) | LTX = LP2000 > EG500 > EFT > SFT > GJ > E5 = FG6 = FGHD | - Transfection efficiency was monitored using flow cytometry and fluorescence microscopy. - LTX and LP2000 had similar cytotoxic effects but the cytotoxicities of 7 other reagents were un-reported. | (Hunt *et al.* 2010) |
| DNA | 11 cells types, namely, P16, REF, MESCs, 16HBE14o-, CFBE41o-, HEK293, SC1 lymphoblasts, LT1-1B1 lymphoblasts, PTE, THE and hematopoietic CD34+ cells | - 3 to 5 x 10^5^ cells and 2 µg pmaxGFP / well were used. - For LP, the transfection reagent (v): DNA(w) ratio were 1/1 to 7/1. - For PEI, the charge ratio of PEI/DNA ranged from 3/1 to 8/1. - For EFT, the transfection reagent (v): DNA(w) ratio ranged from 10/1 to 25/1. - For NF, 1 to 2 x 10^6^ cells were added with 100µL transfection buffer and 2µg/transfection sample. | pmaxGFP plasmid | For **P16**:  NF > PEI > LP2000 > LP+ve>EFT  For **PTE**:  NF > LP2000 > LP+ve > PEI > EFT  For **HTE**:  NF > EFT > LP2000 > PEI > LP+ve  For **HEK293**, nucleofection and Lipofection showed almost similar transfection efficiencies and toxicities. | - Transfection efficiency monitoring using flow cytometry. - Except for HEK293, NF is more superior than chemical transfection in term of efficiency and safety. - Transient transfection showed 40-90% success in all cell types. | (Maurisse *et al.* 2010) |
| DNA | 10 cell types, namely, MC3T3-E1, C3H10T1/2, PT-30, MCF-7, HeLa, C2C12, Hep G2, 4T1, HCT116 and HEK293 | - 2 x 10^5^ cells were transfected inside 96-wells plate. - 1µg of the plasmid DNA was mixed with different transfection ratio (TR)/DNA ratio (w/w), depending on the reagent types. - The incubation duration was 4 hours at 37^0^C. - Cells passage used for the experiment was not described in the study. | Plasmids expressing either luciferase (gWIZ luciferase) or β-galactosidase (gWIZ β-galactosidase) | Transfection efficiency in term of **luciferase expression**  For **MC3YC-E1**:  FGHD > JPI > LP2000 > EF > AI > SFT  For **PT-30**:  AI > FGHD > LP2000 > JPI > SFT > EF  For **C3H10T1/2**:  FGHD > LP2000 > JPI > EF > AI > SFT  For **MCF-7**:  AI > JPI > LP2000 > EF > FGHD > SFT  For **HeLa**:  FGHD > JPI > AI > LP2000 > EF > SFT  For **C2C12**:  FGHD > JPI > AI > EF > SFT > LP2000  For **Hep G2**:  AI > FGHD > JPI > EF > SFT > LP2000  For **4T1**:  AI > FGHD > JPI > SFT > EF > LP2000  For **HCT116**:  AI > FGHD > JPI > EF > LP2000 >SFT  For **HEK**:  AI, EF, FGHD and JPI showed similar trend. LP2000 and SFT showed the weakest transgene effects.  Transfection efficiency in term of β-galactosidase expression  For **MC3T3-E1**:  AI > JPI > EF > FGHD > SFT > LP2000  For **PT-30**:  AI > JPI > SFT > LP2000 > FGHD > ExpressFect  For **C3H10T1/2**:  JPI > FGHD > LP2000 > AI > SFT > EF  For **MCF-7**:  AI > JPI > LP2000 > EF > FGHD > SFT  For **HeLa**:  AI > JPI > EF > FGHD > SFT > LP2000  For **C2C12**:  AI > JPI > FGHD > SFT > LP2000 > EF  For **Hep G2**:  FGHD > AI > JPI > EF > LP2000 > SFT  For **4T1**:  AI > SFT > JPI > EF > FGHD > LP2000  For **HCT116**:  FGHD > AI > LP2000 > JPI > EF > SFT  For **HEK**:  AI > EF > LP2000 > JPI > FGHD > SFT | - Transfection efficiencies monitoring using either luciferase or β-galactosidase expressions. - In some of the studied cell lines, transfection efficiency might be better in the presence of 10% serum as compared to absence of serum. - Cytotoxicity data:   EF = JPI > FGHD > AI > SFT > LP2000   - Combining transfection efficiency and cytotoxicity, FGHD is the most effective transfection reagent, followed by AI and JPI. | (Yamano *et al.* 2010) |
| DNA | Z3 | - 1.8 x 10^4^ cells between passage 10 and 20 were seeded into each well of a 96-wells plate and added with 1 µg/ µL pmaxGFP vector. - The DNA (µg): reagent (µL) ratio differed in different reactions and the reagents used ranged from 1 to 10 µL. - Transfection efficiency and cytotoxicity were assessed at 24 and 48 hours post-transfection. | pmaxGFP vector | XG > JP > LTX > Matra-A | - Fluorescence microscopy was used to assess transfection efficiency. - Comparison of the cytotoxicities:   LTX > XG = JP > Matra-A | (Sandbichler *et al.* 2012) |
| DNA | Rat pheochromocytoma cells from the adrenal medulla | - 4 x 10^4^ cells / well were seeded onto 24-wells plate. - Cells passage ranged from 8-10. The DNA (µg): transfection reagent (µL) ratio was set at 1:3 for all different reagents. - The plasmid amount used was 0.25, 0.5, 0.75 and 1 µg. | DNA plasmid pEGFP-C1 | LP2000 > LTX > TLT1  LP2000 showed higher transfection efficiency than LTX when higher DNA amount was used and the reverse phenomenon was observed when lower DNA amount was used. | - Fluorescence microscopy was used to assess transfection efficiency. - Cytotoxicities comparison:   LP2000 = LTX > TLT1 | (Covello *et al.* 2014) |
| siRNA | bMDM | - 1 x10^5^ cells / well in 24-wells plate were transfected with 50nM siRNA. - The incubation duration was 16 hours before the transfected cells were analysed using flow cytometry. - The bMDM cells were transfected with 0.5 to 10 µL of the different transfection reagents. | siRNAs duplexes | **Comparison of the transfection efficiency:**  IN > XG > DFT3 > HPT > LP2000 > DFT2 > DFT4 > RNAiMAX > DFT1 > N-TER > siPORT Amine  **Comparison of the knock-down effects post-transfection:**  LP2000 > DFT3 > RNAiMAX > IN > XG | - Transfection efficiency monitoring using flow cytometry and qPCR. - Higher concentration of DFT2 and 4 showed higher cytotoxicity. - LP2000, RNAiMAX and DFT3 gave the most effective transfection outcome with considerably lower cytotoxicity effect. | (Jensen *et al.* 2014) |
| DNA | hPDLSCs | - 2 – 3 x 10^4^ hPDLSCs cells / well were seeded into 24-wells plate. - The authors either used same doses, double or halves doses of the reagents and chemicals as described in the manufacturer protocol. - The transfection efficiency was assessed at 24- and 48-hours post-transfection. - Cells at passage 3-5 were used. | pGUP6/GFP/Neo-shNC plasmid | LP2000, PEI, GBfectene-Elite and XG showed poor efficiencies (<6%) in transfecting hPDLSCs as compared to the positive control (lentiviral vectors) (95%)  MATRA-A showed higher transfection efficiency (~11%) | Transfection efficiency was observed using fluorescence microscopy and flow cytometry.  Chemical reagents showed higher cytotoxicity as compared to the magnetic assisted transfection method. | (Wang *et al.* 2015) |
| DNA | HEK293 | - 2.5x10^5^ cells / 2mL, 2.5µg plasmid DNA, 7.5-12.5µL different transfection reagents, 200-250µL OPTI-MEM reagents, 5µL P3000 reagents for reaction involving LP3000. - The incubation time for DNA-lipid complexes varied from 5 to 15 minutes. - The cells were left transfected for overnight. | Expression vector (pcDNA) carrying the DNA of interest (*AtGORK*) | LP3000 > FGHD > LP2000 | - Transfection efficiency monitoring using fluorescence microscopy. - Cytotoxicity of each transfection reagent was unreported. - Early passage cell line was recommended for transfection work. | (Ooi *et al.* 2016) |
| DNA and oligonucleotides (siRNA) | 7 cell types, namely, HEK293, Neuro2a, C2C12 myoblasts, C2C12 myotubes, hTERT MSCs, SMA and HepG2 | - 10^4^ or 5 x 10^4^ cells were used for the transfection in either 96- or 24-wells format. - For LP2000, 1 µg of the nuclei acid materials were mixed with 2.5 µL of the reagent. - The mixing formula for other transfection reagents were not specified in details. | Splice-corrected plasmid containing minicircle DNA and oligonucleotides | RNAiMAX > LP2000 > XG9 > DTP > FG6 > TKO | - Transfection efficiency assessment using fluorescence microscopy, luminescence reporting system and qPCR. - Freeze-thaw of the lipid-based transfection reagents might enhance transfection efficiency, without compromising the cells viability. - Only cells transfected with LP2000 showed cells viability results, but not for cells transfected with other reagents. | (Sork *et al.* 2016) |
| DNA and siRNA | E14 and R1 mESc | - For adherent cultures, 2 x 10^5^ cells / well were seeded in 12-wells plate. - For single cells suspension, 5 x 10^5^ cells / well were used. 1.5 µg of the plasmid DNA was added per well. - For siRNA transfection, both adherent and single cells culture were seeded with 5 x 10^4^ cells / well in 24-wells plate and the final siRNA concentration of 50 to 100nM was added. | pmaxGFP and pCMV plasmids which carried genes encoding β-galactosidase, pCAGGs heparinase vectors and siRNA | Comparison of the **siRNA transfection efficiencies** (based on *Dab2* gene expression):  X2 > NaF > LP3000 > LP2000 > XG > SiQuest > TransIT-TKO  Comparison of the DNA transfection efficiencies in **adherent cultures**:  Xfect > LP2000 > NFN > XG-HP > VF > X2 > JP > LP3000 > FGHD > 2020 > XG-9 > TF > NaF  Comparison of the DNA transfection efficiencies in **single cell suspension**:  Xfect > LP2000 > NaF > X2 > XG-HP > 2020 > JP > LP3000 > TF > VF > FGHD > NF > XG-9 | - Transfection efficiency was monitored using fluorescence microscopy, qPCR, β-galactosidase activity, western blot and flow cytometry. - Comparison of the cell viability in **adherent cultures**:   VF > XG-HP> LP2000> NFN > Xfect   - Comparison of the cell viability in **single cell suspension**:   XG-HP > 2020 > X2 > LP2000 > NaF | (Tamm *et al.* 2016) |
| DNA | HEK293 | - 3 x 10^5^ cells / well were used. - All cells used were below passage 20. - The plasmid DNA: transfection reagent ratio (w/v) was 1µg to 3-4µL. - The transfected cells were incubated for 1 day before harvested for further experiments. | pNL4-3 plasmids and pUltraHot plasmids expressing mCherry | X2 = JP > FGHD > LP2000 | - Transfection efficiency monitoring using flow cytometry. - Cells transfected with LP2000 and FGHD showed reduced level of readable DNA. | (Homann *et al.* 2017) |
| DNA | hBM-MSCs from 6 healthy gentlemen | - 6 x 10^3^ cells / well were seeded onto 96-wells plate in which each well contained 0.3 to 0.6 µL reagents. - The transfection incubation period was 24 hours before measurement of the transfection efficiency. Transfection reagents (v) to DNA (w) ratio ranged from 0.15-0.6 µL to 0.1 µg. | pCMV6-AC-GFP and pCMV6-AC-PIGF | LTX > 2020 > LP3000 > JP > 293 > PEI  2020 is the most effective (~30% efficiency) and safe (~90% viability) transfection reagent in transfecting hBM-MSCs without altering the cells stemness (~95% retention of the cells stemness) | - Transfection efficiency was assessed using fluorescence microscopy and flow cytometry. - Cell viability comparison:   293 > PEI = JP > 2020 > LP3000 > LTX | (Cheung *et al.* 2018) |
| DNA | AGS | - In 24-wells plate, 4-7 x 10^4^ cells / well were seeded. - For AT, the DNA: transfection reagent ratio was 0.2-0.4µg: 0.75-2.25µL. - For XG-HP, the DNA: transfection reagent ratio was 1 µg: 1-4 µL. - The cells-DNA/reagent mixture was incubated for 48 hours before transfection efficiency assessment. | pEGFP1-N1 plasmid | XG-HP > AT | - Transfection efficiency was assessed using fluorescent microscopy and flow cytometry. - Cytotoxicity by AT was <5% compared to 6-9% by XG-HP. | (Gharaati-Far *et al.* 2018) |
| DNA and shRNA | HEK293, HUVECs and mouse cortical neuronal cells | - 2.8-6µg of the different expression plasmids carrying DNA/shRNA, 30µL of LP2000 and 3000 and FG6, 1mL of OPTI-MEM reagents and 30µL for reaction involving LP3000 were used. - Incubation time was 48 hours for all different reaction protocols. - The cell numbers passage prior to the transfection were unclear. | Expression plasmids like lentivirus carrying Cx43 shRNA, plenti CMV GFP, packaging plasmids like pLP/VSVG and pLP1 & 2 plasmids | LP3000 > LP2000 > FG6 | - Transfection efficiency monitoring using fluorescence microscopy and western blot of the downstream target expression (Cx43 protein). | (Shi *et al.* 2018) |
| Single stranded oligonucleotides (SSO) (miRNA) | 10 cell types, U87MG, Huh-7, Hep G2, HEK293, HL-60, MCF-7, MDA-MB-231, SH-SY5Y, JU77, primary myoblast cells | - All transfections were conducted in 24-wells plate with cells density ranged from 5 to 10 x 10^4^ cells / 1mL. - Reaction mixture: - RNAiMAX, the reaction involved mixing of 1µg of SSO with 2 µL of RNAiMAX diluted with OPTI-MEM reagent. - For LP3000, the reaction involved mixing of 1 µg SSO with 2 µL LP3000 and 1.5 µL P3000 reagent in the ratio of 2:1 (transfection reagent (TR): SSO). - LF and LP2000 had the similar mixing formula as LP3000 except that P3000 reagent was excluded. - For FGHD reagent, the mixing formula was 1 µg SSO with 2 µL FGHD reagent in the TR:SSO ratio of 2:1. - The transfection incubation duration for all protocols was 24 hours. | Single stranded oligonucleotide (SSO) with 5’ end labelled with FAM | For **Huh-7**:  FGHD = RNAiMAX > LP3000 > LF > Lipofectamine 2000  For **U87MG**:  LP3000 > FGHD > LP2000 > LF > RNAiMAX  For **MDA-MB-231**:  LP3000 > RNAiMAX > FGHD > LP2000 > LF  For **SHSY5Y**:  LP3000 > RNAiMAX > FGHD > LF > LP2000  For **HepG2**:  FGHD > LP3000 = RNAiMAX > LP2000 > LF  For **JU77**:  LP3000 > LP2000 > RNAiMAX > FGHD > LF  For **HL-60**:  LP2000 > LP3000 > FGHD > RNAiMAX > LF  For **primary myoblast cells**:  LP3000 > RNAiMAX > FGHD > LP2000 > LF  For **HEK293**:  LP3000 > FGHD > LP2000 > RNAiMAX > LF  For **MCF-7**:  LP3000 > LP2000 > RNAiMAX > FGHD > LF  **Overall comparison**:  LP3000 > RNAiMAX = FGHD > LP2000 > LF | - Transfection efficiency was monitored using fluorescence microscopy and qPCR of miR21.   **Relative cytotoxicities:**  LP2000 > LP3000 > FGHD > RNAiMAX > LF   - For MDA-MB-231:   The TR:SSO ratio of 1:1 is the ideal ratio to achieve highest transfection efficiency when LP3000 and RNAiMAX were used.   - For FGHD, 2:1 ratio is the best transfection ratio. - For LP2000 and LF, the TR:SSO ratio of 1:1, 2:1 or 4:1 did not play significant role in affecting the transfection efficiencies. - For all cell types:   The use of LP3000 and P3000 reagents would greatly enhance transfection efficiency as compared to the use of LP3000 alone. | (Wang *et al.* 2018) |
| DNA | hiPSC-CMS | - All transfections were conducted in 12-wells plate with cells density of 2 x 10^5^ cells / 1mL. - For transfection using lipoplexes, the DNA (μg) to reagents (μl) ratio ranged from 0.4-1.3: 0.75-1.2. - For transfection involving TR5 polyplexes, the DNA (μg) to reagents (μl) ratio was 1:1 to 3.1. - For PEI transfection, the PEI nitrogen per DNA phosphate (N/P) ratio used was ranged from 3:1 to 9:1. | pGFP | LP STEM > TR5 > PEI25 > LP3000 > LP2000 | - Transfection efficiency was assessed using flow cytometry.   **Relative cytotoxicities:**  LP2000 = LP3000 = LP STEM > TR5 > PEI25  LP STEM is the best reagent for transfecting hiPSC-CMS which produced highest transfection efficiency and cell viability | (Tan *et al.* 2019) |

**Cell lines details:** AGS: Human gastric adenocarcinoma cell line; A549: Human adenocarcinomic alveolar basal epithelial cell; A673: Human ewing sarcoma cell line; A-10 SMCs: Rats smooth muscle cells; bMDM: Bovine monocyte-derived macrophages; C2C12: Mouse myoblasts; C3H10T1/2: Mouse stem cells; HAECs: Human aorta endothelial cells; HASMCs: Human aorta smooth muscle cells; hBM-MSC: Human bone marrow mesenchymal stem cells; HCT116: Human colorectal carcinoma cell line; HEK: Primary human epidermal keratinocytes; HEK293: Human embryonic kidney cells 293; HeLa: Human cervical cancer cell; Hep G2: Human hepatocellular carcinoma cells; hiPSC-CMS: human induced pluripotent stem cells derived cardiomyocytes; HL-60: Human leukemia cell line; hPDLSCs: Human periodontal-ligament stem cells; hTERT MSCs: Human telomerase reverse transcriptase mesenchymal stem cells; Huh-7: Human liver cell line; HUVECs: Human umbilical cord vein cells; JU77: Human primary myoblast and human lung mesothelioma cells; MCF-7: Oestrogen responsive breast cancer cell line; MC3T3-E1: Mouse preosteoblasts; MDA-MB-231: Triple negative breast cancer cell line; MESCs: Sickle cell disease transgenic mice embryonic stem cell; Neuro2a: mice neuroblastoma cells; Panc 04.03: Human pancreas epithelial cancer cell line; PECs: Primary endothelial cells; PTE and THE: Primary pig and human tracheal epithelial cells; PT-30: Human epithelial precancer cells ; P16: Primary embryonic pig fibroblasts; REF: Embryonic rabbit ear fibroblasts; SH-SY5Y: Human neuroblastoma cells; SKOV3: Human ovarian cancer cell line; SMA: Spinal muscular atrophy fibroblast cells; UMR: Rat osteosarcoma cell line; U87MG: human glioblastoma cells ; Z3: Embryonic zebrafish cell line; 4T1: Mouse mammary carcinoma; 16HBE14o- and CFBE41o-: Immortalized human bronchial epithelial cells. **Abbreviations for transfection related chemicals/methods:** AT: Attractene; DAC: 3β-[*N*-(*N*,*N*’-dimethylaminoethane)-carbamoyl]; DC: 3β-[(*N*,*N*’-dimethylaminoethane)-carbamoyl]; DFT1: DharmaFECT1; DFT2: DharmaFECT2; DFT3: DharmaFECT3; DFT4: DharmaFECT4; DOTAP: DTP; EF: ExpressFect; EFT: Effectene; EG500: ExGen 500; E5: Escort IV; FGHD: FuGENE HD; FG6: FuGENE 6; GJ: GeneJammer; HPT: HiperFect; IN: INTERFERin; JP: JetPrime; JPI: JetPEI; LF: Lipofectin; LP+ve: Lipofectamine plus; LP2000: Lipofectamine 2000; LP3000: Lipofectamine 3000; LTX: Lipofectamine LTX; Matra-A: Magnetic-assisted transfection; NaF: Nanofectamine; NFN: Nanofectin; NF: Nucleofection; OF: Oligofectamine; PEI: Polyethylenimine; RNAiMAX: Lipofectamine RNAiMAX; SFT: SuperFect; TF: TurboFect; TKO: TransIT-TKO; TLT1: TransIT-LT1; VF: ViaFect; XG: X-tremeGENE; XG-HD: X-tremeGENE-HD; XG-9: X-tremeGENE-9; X2: TransIT-X2; 293: TransIT-293; 2020: TransIT-2020; “=”: Similar; “>”: More superior.
